# Gamithromycin in swine: Pharmacokinetics and clinical evaluation against swine respiratory disease

**DOI:** 10.1002/vms3.375

**Published:** 2020-10-15

**Authors:** Dietmar Hamel, Alexandra Richard‐Mazet, Florian Voisin, Inge Böhne, Florence Fraisse, Renate Rauh, Rose Huang, Michael Kellermann, Laura Letendre, Pascal Dumont, Steffen Rehbein

**Affiliations:** ^1^ Boehringer Ingelheim Vetmedica GmbH Rohrdorf Germany; ^2^ Boehringer Ingelheim Animal Health France Lyon France; ^3^ Hyovet Plestan France; ^4^ Tierartzpraxis Böhne Melle‐Wellingholzhausen Germany; ^5^ Boehringer Ingelheim Animal Health USA, Inc. North Brunswick NJ USA

**Keywords:** clinical efficacy, gamithromycin, pharmacokinetics, respiratory disease, swine

## Abstract

The pharmacokinetics of gamithromycin were evaluated in 26 male castrated and female crossbred swine administered gamithromycin 15% w/v (Zactran®, Boehringer Ingelheim) intravenously at 6 mg/kg bodyweight or intramuscularly at 3, 6 or 12 mg/kg bodyweight. Blood samples were collected up to Day 10 to establish the plasma profile of gamithromycin, bioavailability and dose proportionality. When administered by intramuscular injection at 6 mg/kg BWT, pharmacokinetic parameters were as follows: area under the curve until last quantifiable plasma concentration, 5.13 ± 0.957 µg*hours/ml; maximum plasma concentration, 960 ± 153 ng/ml at 5 to 15 min; terminal half‐life of 94.1 ± 20.4 hr. Absolute bioavailability was 92.2%. Increase in systemic exposure was proportional to the gamithromycin dose level over the range 3–12 mg/kg BWT. No gender‐related statistically significant difference in exposure was observed. For clinical evaluation of Zactran® against swine respiratory disease, 305 pigs from six commercial farms in three countries in Europe with signs associated with *Actinobacillus pleuropneumoniae* and/or *Haemophilus parasuis* and/or *Pasteurella multocida* and/or *Bordetella bronchiseptica* were used. At each site, animals were treated once in a 1:1 ratio with a single intramuscular dose of Zactran® (6 mg gamithromycin/kg bodyweight) or Zuprevo® (4% w/v tildipirosin at 4 mg/kg bodyweight; MSD Animal Health) at the recommended dose respectively. Animals were observed and scored daily for 10 consecutive days for signs of swine respiratory disease (depression, respiration and rectal temperature), and animals presenting signs of clinical swine respiratory disease (Depression Score 3 and/or Respiratory Score 3 associated with Rectal Temperature > 40.0°C) were removed from the study and considered as treatment failure. Animals which remained in the study were individually assessed for ʽtreatment successʼ or ʽtreatment failureʼ (Depression Score ≥ 1 and Rectal Temperature > 40.0°C or Respiratory Score ≥ 1 and Rectal Temperature > 40.0°C). Using a non‐inferiority hypothesis test (non‐inferiority margin = 0.10), the proportion of treatment successes in the Zactran® group (97%) was equivalent to or better than that in the Zuprevo® group (93%).

## Graphical abstract

Intramuscular injection of gamithromycin 15% w/v in swine at 6 mg/kg is characterized by rapid absorption and distribution to tissues. Clinical evaluation in an European field study incorporating 305 swine has shown safe and efficacious treatment for swine with bacterial respiratory disease.

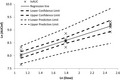

## 1 INTRODUCTION

Gamithromycin (GAM) belongs to the 15‐membered semisynthetic macrolide antibiotics and, as an azalide, is characterized by a uniquely positioned alkylated nitrogen at 7a‐position of the lactone ring.

Macrolides display bacteriostatic action by inhibiting bacterial protein biosynthesis (Jain & Danzinger, 2004; Menninger, 1985; Retsema, 1999) and accumulate in host defence cells and extracellular fluid (Matteos & Nightingale, 2002), thus, reaching more than therapeutic concentrations at the host–pathogen interface. Previous studies in cattle reported that GAM is rapidly absorbed and distributed to highly perfused organs, and reaches high and prolonged concentrations in the lungs (Bryskier & Bergogne‐Berezin, 1999; Giguère et al., 2011; Huang et al., 2010).

Similar to its efficacy in the treatment and control of bacterial bovine respiratory disease following a single subcutaneous injection with GAM 15% w/v (Baggott et al., 2011; Linhardt & Brumbaugh, 2019; Torres et al., 2017), GAM should display favourable characteristics in the treatment of clinical swine respiratory disease. This multifactorial condition results from mixed infection of viral and/or bacterial agents and is characterized by anorexia, fever (>40°C), cough and dyspnoea, ultimately leading to decreased feed conversion and growth rate (Opriessnig et al., 2011).

Gamithromycin 15% w/v injection (Zactran®, Boehringer Ingelheim) is currently licensed in Europe, the Americas and other regions for the treatment and control of bovine respiratory disease in cattle caused by *Mannheimia haemolytica*, *Pasteurella multocida* and *Histophilus somni,* and/or swine respiratory disease caused by *Actinobacillus pleuropneumoniae, P. multocida, Haemophilus parasuis* and *Bordetella bronchiseptica*. In addition, the product was licensed in Europe for the treatment of footrot in sheep caused by *Dichelobacter nodosus* and *Fusobacterium nodosus* (EMA, 2018a).

This study describes the results of studies on the pharmacokinetic profile of GAM in swine following intravenous and intramuscular (IM) injection and reports results of an European multicentre field study on the clinical evaluation of a single GAM 15% w/v injection at a dose of 6 mg/kg bodyweight (BWT) IM against swine respiratory disease.

## 2 MATERIALS AND METHODS

### 2.1 General study design

The pharmacokinetic study was conducted in accordance to GLP and to “Guidelines for the Conduct of Pharmacokinetic Studies in Target Animal Species, EMEA/CVMP/133/99‐FINAL”. The European multicentre field study was in accordance with the Committee for Veterinary Medicinal Products “Guideline on Good Clinical Practice – VICH Topic GL9 GCP, CVMP/VICH/595/98‐Final” and the “Guideline for the demonstration of efficacy for veterinary medicinal products containing antimicrobial substances, EMEA/CVMP/627/01‐FINAL”. All study procedures complied with the appropriate local animal welfare regulations, and were approved by applicable legal bodies and by the company´s animal welfare committees. The on‐farm procedures in the field study were performed with the informed consent of the animal owners.

### 2.2 Pharmacokinetic study

Twenty‐eight (14 male castrated, 14 female) healthy German Landrace x Pietrain pigs, aged approximately 3.4 months were used. None of the swine had received any macrolide antibiotic treatment within 1 month prior to Day 0 (=day of treatment). All animals were fitted with two surgically implanted jugular vein catheters 1 week prior to treatment. The animals were allocated to treatment groups based on Day −1 BWT; the heaviest pig within each sex was selected to form the non‐treated control group (Group 1), the remaining animals were ranked by decreasing BWT within sex and formed in three blocks of five, four and four animals, and were randomly allocated to Groups 2, 3, 4 and 5 consisting of total eight, six, six and six animals, respectively. Gamithromycin 15% w/v was administered once on Day 0 either by intravenous (IV) (Group 2) or by IM injection (Groups 3, 4 and 5) based on Day −1 BWT. Animal and dosing details are given in Table 1.

**TABLE 1:** Animal and treatment details, pharmacokinetic study

Group	Number of animals, sex^a^	Body weight ([BWT,]kg)^b^	Dose of Gamithromycin (15% w/v)	GAM (mg/kg BWT)
1	2 (1 mc, 1 f)	50.6, 54.6	Untreated control	Not applicable
2	8 (4 mc, 4 f)	43.8–51.2	0.2 ml/5 kg BWT^c^, IV	6
3	6 (3 mc, 3 f)	44.2–51.4	0.1 ml/5 kg BWT^c^, IM	3
4	6 (3 mc, 3 f)	44.6–50.0	0.2 ml/5 kg BWT^c^, IM	6
5	6 (3 mc, 3 f)	51.8–50.6	0.4 ml/5 kg BWT^d^, IM	12

^a^ mc = male castrate; f = female; IV = intravenous; IM = intramuscular

^b^ Day‐1 body weight used for dose calculation purposes.

^c^ The calculated dose was rounded up to the next 0.1 ml increment, if it was not an exact 0.1 ml increment.

^d^ The calculated dose was rounded up to the next 0.2 ml increment, if it was not an exact 0.2 ml increment.

Gamithromycin 15% w/v was given to Group 2 pigs via one IV catheter; following administration, the catheter was flushed with ~20 ml of physiological saline solution to ensure delivery of the targeted dose. In animals of Groups 3, 4 and 5, GAM 15% w/v was administered IM in the dorsal part of the left neck side, in front of the shoulder.

Gamithromycin 15% w/v was administered using 0.1 ml graduated syringes in Groups 2, 3 and 4 or using 0.2 ml graduated syringes in Group 5. Intramuscular doses were administered with sterile disposable hypodermic 19 G x 1’’ needles.

Following GAM 15% w/v administration, the animals were observed hourly for the first four hours for reactions to treatment. Animals were housed individually in pens with straw bedding and they were fed a complete fattening ration offered for ad libitum consumption and had free access to water.

### 2.3 Collection of plasma samples

Whole blood was collected from the jugular vein via the catheters into lithium heparinized tubes prior to treatment (Day −1) from all animals and from Group 1 animals on Days 5 and 10. Groups 2 to 5 animals were sampled at 5 (± 2), 10 (± 2), 15 (± 5) and 30 (± 5) minutes, 1, 2, 3, 6, 10 and 24 hr (± 20 min) after treatment, and on Days 2, 3, 4, 5, 6, 7, 8, 9 and 10 (within 1 hr of the Day 0 treatment time). Prior to blood sampling, catheters were flushed with physiological saline solution. The first ~5 ml of blood was discarded, and thereafter, blood for plasma processing was collected. Following blood collection, catheters were flushed with ~10 ml of physiological saline solution and a ~5 ml bolus of anticoagulant in saline was placed in the catheter. Plasma was separated by centrifugation and stored at ≤‐20°C until assayed for GAM concentrations.

### 2.4 Analytical method

Plasma samples were analysed for GAM using a validated LC‐MS/MS method (Huang et al., 2010). The lower limit of quantitation in plasma was established as 2.0 ng/ml and the lower limit of detection as 1.0 ng/ml. The method performed well during the analysis of the samples. Quality control samples had GAM recoveries from 85%–114% (mean 102 ± 5%).

### 2.5 Pharmacokinetic analysis

Pharmacokinetic analysis was performed using a non‐compartmental model with the linear up/down trapezoidal model using WinNonLin® version 5.0.1 (Pharsight Corporation, Mountain View, CA, USA) for each individual animal, and parameters were averaged for the group. Only GAM plasma concentrations above the lower limit of quantitation were included in the pharmacokinetic analysis.

The maximum concentration and observed time to maximum concentration, and time to last quantifiable concentration were determined directly from plasma concentration data. The first‐order rate constant λ_z_, associated with the terminal log‐linear portion of the curve was estimated via linear regression of the log drug plasma concentration versus time curve, and the terminal plasma half‐life (T_1/2_) concentration was calculated using T_1/2_ = ln(2)/ λ_z_. The area under the curve (AUC) was determined using the linear trapezoidal rule for increasing and the logarithmic trapezoidal model for decreasing plasma concentrations from Day 0 to the last time plasma drug concentrations were above the lower limit of quantitation (AUC_last_). AUCs were extrapolated to infinity using the formula AUC_inf_ = AUC_last_ + C_last_/λ_z_.

Dose proportionality of GAM following IM administration over the range 3–12 mg/kg BWT was assessed by analysing the dose‐normalized average AUC_inf_ at each dose level from Groups 3, 4 and 5 and also using a power model:

$$\text{Ln}\left({\text{AUC}}_{\text{inf}}\right)=\beta 0+\beta 1\text{Ln}\left(\text{Dose}\right).$$

Linear regression analysis was performed using Proc Reg in SAS 9.0 (SAS Corporation, Cary, NC, USA) with weighting of (Dose)‐1. The residuals were normally distributed, independent and randomly distributed around zero. The 95% confidence limits on the parameters were determined at α = 0.05.

A *t* test was utilized to determine statistical differences between the pharmacokinetics of male castrate and female swine.

### 2.6 European multicentre field study

Various commercial cross‐breed pigs were used in this study conducted in six commercial fattening farms located in France, Germany and Spain (Table 2). Only swine respiratory disease‐positive animals from available stock were eligible for enrolment. At each site, animals were housed in group pens, study animals together with non‐study animals, within one airspace. They were managed according to the normal husbandry practices at each site and fed according to local practice ensuring adequate nutrients for their age and condition. Routine disease control measures were similar for all animals at a study site and were limited to the administration of (but not necessarily any or all of) viral vaccines and endectocides. None of the animals had received bacterial vaccines against swine respiratory disease pathogens (*A. pleuropneumoniae*, *P. multocida*, *B. bronchiseptica* and *H. parasuis*). Animals at three of the six sites were vaccinated with *Mycoplasma hyopneumoniae* vaccines. None of the animals had received medications that potentially impact treatment response (e.g. systemic corticosteroids, NSAIDs, other systemic antimicrobials) within 15 days of enrolment. For farm qualification, the first three pigs meeting inclusion criteria on each site served as swine respiratory disease sentinels for further diagnosis (see Enrolment).

**TABLE 2:** Animal and treatment details, multicentre field study

Farm	Breed^a^	Age (weeks)	Body weight (kg)^b^	Number enrolled and treated		Number included in efficacy analysis	
				Zactran^c^	Zuprevo^d^	Zactran^®^	Zuprevo^®^
France 1	Pie x Naïma	~17	40.8–86.0	35	35	35	35
France 2	LW x LR x Pie	~17	32.6–75.6	28	28	28	27
France 3	LW x LR x Pie	~15	37.0–83.0	31	31	28	31
France 4	Pie x LW	~16	34.5–76.5	13	13	13	13
Germany	LW x LR x Pie	~11	22.5–43.5	34	34	34	34
Spain^e^	LW x LR x Pie	~14	20.5–38.7	12	11	12	11

^a^ Pie = Pietrain; LW = Large White; LR = Landrace

^b^ Animals were weighed on Day 0 on verified scales for dose calculation purpose.

^c^ Single dose of gamithromycin 15% w/v at 1 ml per 25 kg body weight (equivalent to 6 mg/kg).

^d^ Single dose of tildipirosin 4% w/v at 1 ml per 10 kg body weight (equivalent to 4 mg/kg).

^e^ Three animals were enrolled on one occasion.

Day 0 (= day of treatment) varied by site and by animal. Upon enrolment, animals were weighed and allocated randomly by means of site‐specific randomization lists to treatments in a 1:1 ratio: Treatment Group 1, GAM 15% w/v (Zactran®); Treatment Group 2, tildipirosin (TIL) 4% w/v (Zuprevo®) in blocks of two animals. Treatments (commercial doses) were administered as described in Table 2. The doses were rounded up to the next 0.1 ml increment for doses up to 2 ml and were rounded up to the next 0.2 ml increment for doses >2 ml. Treatments were administered IM on the dorsal left part of the neck. For Zuprevo®, total doses of >5 ml were divided into 5 ml (primary injection) and the remainder (secondary injection on the dorsal right part of the neck).

Animals in a block were treated the same day. Enrolment at each site was performed for up to 3 consecutive days after enrolment of the first block. Animals of both treatment groups were housed comingled at all sites.

### 2.7 Enrolment

Animals were scored for clinical signs of swine respiratory disease based on depression and respiratory signs as given in Table 3 and rectal temperature. Animals meeting the inclusion criteria (Depression Score ≥2, Respiratory Score ≥2 and Rectal Temperature >40.0°C) were eligible for enrolment. The first three animals at each site fulfilling the inclusion criteria served as swine respiratory disease sentinels and were sampled by bronchoalveolar lavage or nasal swabs, euthanized and necropsied to obtain lung and other tissue (tonsil and lymph nodes) samples for bacterial culture and/or PCR. To further support qualification of the farm for the study, nasal swabs or bronchoalveolar lavages were collected from each enrolled study animal on Day 0 prior to treatment.

**TABLE 3:** Scoring for clinical SRD signs, multicentre field study

Sign	Score	Description
Depression	0	Normal: alert, active normal appetite, well hydrated, coat normal
	1	Mild: moves slower than normal, slightly rough coat, may appear lethargic but upon stimulation appears normal
	2	Moderate: inactive, may be recumbent but is able to stand, gaunt, may be dehydrated
	3	Severe: down or reluctant to get up, gauntness evident, dehydrated
Respiratory	0	Normal: rate and pattern normal, no abnormal nasal discharge
	1	Mild: slightly increased respiratory rate, some roughness in breathing.
	2	Moderate: increased respiratory rate, some abdominal breathing
	3	Severe: increased respiratory rate with abnormal effort – open mouth breathing, grunting, dog sitting

Nasal swabs, bronchoalveolar lavages and/or tissue samples (from sentinels and animals removed from the study) were subjected to standard culturing methods for isolation and speciation of *A. pleuropneumoniae*, *P. multocida*, *B. bronchiseptica* and *H. parasuis*.

### 2.8 Follow‐up

Study animals were observed and scored daily from Day 0 to Day 10 inclusive for signs of clinical swine respiratory disease. Animals meeting removal criteria (Depression Score = 3 and/or Respiratory Score = 3, associated with Rectal Temperature ≥40.0°C) were removed from the study, euthanized and necropsied for confirmation of swine respiratory disease. Animals developing a non‐swine respiratory disease concurrent pathological condition were also removed from the study.

Personnel involved with post‐treatment evaluations were masked; they were not present during treatments and did not have access to the allocation/treatment assignments.

### 2.9 General and local tolerance

The general reactions to treatments were assessed daily from Day 0 until Day 10 inclusive. The injection sites were observed approximately 1 hr post‐treatment, on the day of removal or at final assessment on Day 10, as applicable. In the event that injection site reactions were present on Day 0, the injection site was observed daily until resolution.

### 2.10 Clinical evaluation and data analysis

All animals removed from the study according to swine respiratory disease removal criteria and confirmed swine respiratory disease positive by necropsy were included in the efficacy analysis and considered as treatment failures. Animals removed for non‐swine respiratory disease pathological conditions were not included in the data analysis.

On Day 10, each remaining animal was assessed and as overall clinical evaluation considered as “Treatment Success” (defined as clinical cure) or “Treatment Failure” (= Depression Score ≥1 and Rectal Temperature >40.0°C or Respiratory Score ≥1 and Rectal Temperature >40.0°C). For analysis of individual clinical signs of swine respiratory disease, Depression Score and Respiratory Score were dichotomized as “Acceptable” (0 or 1) and “Not acceptable” (>1). Rectal temperatures were also dichotomized as “Acceptable” (≤40.0°C) and “Not acceptable” (>40.0°C). Data from all six sites were combined for analysis. The percentage of treatment success and each clinical parameter were compared between groups using a non‐inferiority hypothesis test (non‐inferiority margin = 0.10) (SAS® 9.1.3, Cary, NC, USA). The hypothesis of non‐inferiority between the two Treatment Groups was tested for the proportion of treatment success, and the proportions of acceptable Depression Score, Respiratory Score and Rectal Temperature.

## 3 RESULTS

### 3.1 Pharmacokinetic study

A summary of the basic pharmacokinetic parameters is given in Table 4.

**TABLE 4:** Summary of basic pharmacokinetic parameters of gamithromycin in plasma of swine, pharmacokinetic study

Parameter	Group Mean ± Standard Deviation			
Route of administration	IV	IM	IM	IM
Number of animals in group	8	6	6	6
Gamithromycin dose (mg/kg)	6	3	6	12
AUC_last_ (hr*µg/ml)	5.65 ± 0.712	2.90 ± 0.700	5.13 ± 0.957	9.35 ± 1.32
AUC_inf_ (hr*µg/mL)	5.89 ± 0.658	3.15 ± 0.696	5.43 ± 0.949	9.60 ± 1.27
C_max_ (ng/mL)	—	843 ± 412	960 ± 153	1,390 ± 442
T_max_ (hr)	—	0.083 to 0.250	0.083 to 0.250	0.083 to 0.250
T_1/2_ (hr)	76.1 ± 23.7	79.5 ± 24.3	94.1 ± 20.4	74.2 ± 15.0
V_ss_ (L/kg)	39.2 ± 12.4	—	—	—
Cl_obs_ (mL/h/kg)	1,030 ± 125	—	—	—
*F* (%)	—	107%	92.2%	81.4%

Note

IV = intravenous; IM = intramuscular; AUC_last_ = area under the curve (AUC) from Day 0 to the last time plasma drug concentrations were above LOQ; AUC_inf_ = AUC extrapolated to infinity; C_max_ = maximum concentration, T_max_ = time to maximum concentration; T_1/2_ = half‐life; V_ss_ = volume of distribution at steady state; Cl_obs_ = observed clearance; *F* = Bioavailability

Following a single IV injection of GAM at 6 mg/kg BWT, the AUC_inf_ was 5.89 ± 0.658 µg*hr/ml and the T_1/2_ was 76.1 ± 23.7 hr. The full AUCs were captured in this study with less than 10% extrapolated. The volume of distribution at steady state and clearance were 39.2 ± 12.4 L/kg and 1,030 ± 125 ml h^−1^ kg^−1^ respectively.

For animals treated with IM injection of GAM at 6 mg/kg BWT, the AUC_inf_ was 5.43 ± 0.949 µg*hr/ml which is comparable with the AUC_inf_ following the same dose given IV, resulting in 92.2% absolute bioavailability. The AUC_inf_ for IM injections of 3 and 12 mg/kg were 3.15 ± 0.696 and 9.60 ± 1.27 hr*µg/ml respectively. The maximum concentrations were 843 ± 412, 960 ± 153 and 1,390 ± 442 ng/ml for IM GAM doses of 3, 6 and 12 mg/kg respectively. The observed time to maximum concentration in the three IM injection groups was observed between 5 and 15 min post dose. The T_1/2_ following IM injection was 79.5 ± 24.3, 94.1 ± 20.4 and 74.2 ± 15.0 hr, respectively, for the animals dosed GAM at 3, 6 and 12 mg/kg BWT, which is comparable with T_1/2_ following IV administration (76.1 ± 23.7 hr). High systemic bioavailability (81.4% to 107%) was observed for all dose levels administered via the IM route of administration.

**FIGURE 1:**
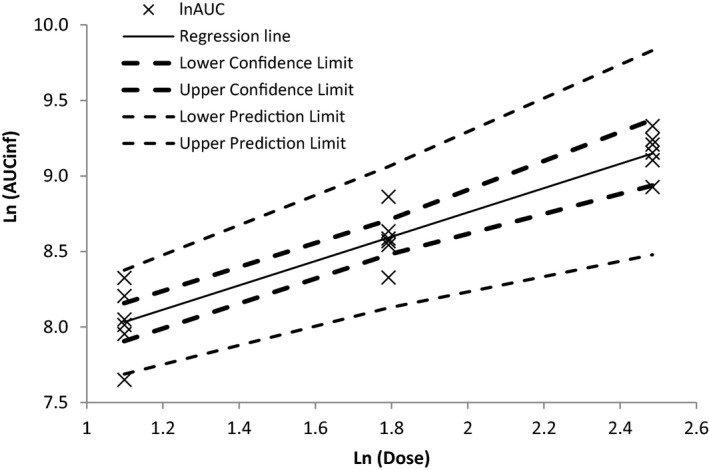
Gamithromycin Log AUC_inf_ (hr*µg/ml) versus Log Dose (mg/kg) following intramuscular administration of gamithomycin 15% w/v (Zactran^®^) at 3, 6 and 12 mg per kg body weight to swine

The dose normalized average maximum concentrations were 281 ± 137, 160 ± 26 and 116 ± 37 (ng/ml)/(mg/kg) for the 3, 6 and 12 mg/kg doses, respectively, indicating that the average maximum concentration values did not increase proportionally with dose. The dose normalized average AUC_inf_ was 1.05 ± 0.23, 0.91 ± 0.16 and 0.80 ± 0.11 (day*ng/ml)/(mg/kg) for the 3, 6 and 12 mg/kg doses respectively. There was no statistical difference between the 6 mg/kg target dose normalized AUCs and the 0.5 or 2 times dose normalized AUCs (Student's T‐test, 95% confidence), indicating that the average AUC_inf_ values increased proportionally with dose. Dose proportionality (Figure 1) was also assessed based on regression of AUC_inf_ versus dose at 0.5, 1 and 2 times of the target dose rate with the following resulting equation:

$$\text{Ln}\left({\text{AUC}}_{\text{inf}}\right)=7.1420+0.8102\text{Ln}\left(\text{Dose}\right)$$

The coefficient of determination was 0.8277, and the slope and intercept were highly statistically significant (*p* < .0001) and, therefore, not equal to zero. The mean value for the slope was 0.8102.

There was no statistical difference between T_1/2_ and the AUCs of male castrate and female swine (Student's T test, 95% confidence), indicating that there was no gender‐related difference in the pharmacokinetics.

No abnormal health observation or adverse experience occurred during the study except in one animal in Group 2. The animal exhibited 40.6°C rectal temperature, increased respiration and tachycardia in the afternoon of Day 2 and received anti‐inflammatory treatment (meloxicam) for 3 days. The condition had improved on Day 3, and the animal was normal at Day 6.

### 3.2 Multicentre field study

All 18 sentinels (*i.e*. three per site) presented pathological changes of the lungs (*e.g*. hepatization, congestive area, pneumonia, pleuritis) consistent with swine respiratory disease. At all six farms, presence of bacterial swine respiratory disease pathogens was confirmed from samples collected at necropsy and/or nasal swabs or bronchoalveolar lavages collected from the study animals at enrolment: *B. bronchiseptica* and *P. multocida* at all farms; *A. pleuropneumoniae* and *H. parasuis* in all farms but the farm in Germany.

No study animal died during the study and no adverse events occurred that were considered to be related to treatment. Six of the 153 GAM‐treated animals and 10 of the 152 TIL‐treated animals presented visible injection site reactions characterized as localized firm swelling and heat on touch at the site of injection on the day of treatment. These reactions resolved rapidly within 1 day and no other injection site reactions were recorded in any of the study animals.

Due to clinical manifestations of swine respiratory disease, three GAM‐treated animals and five TIL‐treated animals were removed from the study. For non‐swine respiratory disease reasons, four animals were removed from the study: three GAM‐treated animals on animal welfare grounds; one TIL‐treated animal because of unintentional underdosing. Non‐swine respiratory disease‐related removals were excluded from the data analysis, whereas swine respiratory disease‐related removals remained eligible for analysis. Consequently, a total of 301 pigs were included in the analysis, 150 GAM‐treated animals and 151 TIL‐treated animals.

Results of the data analysis are summarized in Table 5. The proportion of treatment successes was 97% in the GAM group and 93% in the TIL group. As the lower limit of the two‐sided 95% confidence interval on the difference was greater than the non‐inferiority threshold, data supported that GAM treatment was comparable with or better than TIL treatment.

**TABLE 5:** Analysis of clinical evaluation data of gamithromycin versus tildipirosin in the treatment of SRD

Parameter	GAM 15% w/v (Zactran^®^) at 6 mg/kg body weight % (*n* “success” or “acceptable”, total *n*)^a^	Tildipirosin 4% w/v (Zuprevo^®^) at 4 mg/kg body weight % (*n* “success” or “acceptable”, total *n*)	95% confidence interval^b^		Non‐ inferiority Threshold^c^
			lower	upper	
Overall Evaluation^d^	97% (145/150)	93% (141/151)	−0.02	0.08	−0.10
Depression Score^e^	95% (143/150)	95% (144/151)	−0.05	0.05	−0.10
Respiration Score^f^	93% (139/150)	94% (142/151)	−0.07	0.04	−0.10
Rectal Temperature^g^	93% (139/150)	91% (137/151)	−0.04	0.08	−0.10

^a^ The percentage of animals that had an acceptable outcome for the listed endpoint (“success” for Overall Evaluation and “acceptable” for Depression Score, Respiration Score and Rectal Temperature). The numbers in parentheses are the number of animals with acceptable outcome over total number of animals in the group which were included in the analysis.

^b^ Two‐sided 95% confidence interval was computed using the formula, (P_1_‐P_2_)±1.960√{(1/n_1_ + 1/n_2_)P_AVE_ (1‐P_AVE_)}, where P_1_ and n_1_ are the proportion of acceptable responses and number of animals treated for the Zactran® group, P_2_ and n_2_ are the proportion of acceptable responses and number of animals treated for the Zuprevo® group, 1.960 is the 97.5th percentile for the standard normal probability distribution and P_AVE_ is the weight average of the two proportions with weights the number of animals within the treatment group.

^c^ If the lower CI limit is > the non‐inferiority threshold, then for that clinical endpoint, the hypothesis that Zactran® is inferior to Zuprevo® is rejected and the data support that the two treatments are comparable.

^d^ On day 10, an animal was defined a failure if Depression Score ≥ 1 OR Respiratory Score ≥ 1 AND Rectal Temperature > 40°C.

^e^ On day 10, Depression Scores of 0 and 1 were defined as “acceptable”.

^f^ On day 10, Respiration Scores of 0 and 1 were defined as “acceptable”.

^g^ On day 10, Rectal Temperatures of ≤ 40°C were defined as “acceptable”.

Individual analysis of the proportions of acceptable Depression Score, Respiratory Score and Rectal Temperature demonstrated that the GAM group was comparable with or better than the TIL group (acceptable Depression Score 95% in both groups; acceptable Respiratory Score 93% and 94% and acceptable Rectal Temperature 93% and 91% respectively).

## 4 DISCUSSION

### 4.1 Pharmacokinetic study

Based on the results of this study, GAM administered once to swine as 15% w/v injectable solution by IM route at three different doses demonstrated high bioavailability, fast absorption, rapid and extensive distribution to tissues, high clearance and approximate dose proportionality of AUC_inf_.

The pharmacokinetic profile of GAM following IM administration at 6 mg/kg BWT in the present study was largely similar to that established by Wyns et al. (2014), indicating that the pharmacokinetic profile in swine is predictable and consistent across studies despite different size of the animals and different route of administration.

GAM has overall a comparable pharmacokinetic profile across several species and irrespective of the route of administration as shown in domestic animals other than swine including cattle (DeDonder et al., 2016; Giguère et al., 2011; Huang et al., 2010), sheep (Kellermann et al., 2014), young foals (Berghaus et al., 2012; Berlin et al., 2014) and poultry (Watteyn et al., 2013; 2015) (Table 6).

**TABLE 6:** Basic pharmacokinetic parameters of gamithromycin in various animal species

Species (number, gender, age, bodyweight [BWT])	No. per Group	Dose, Route	AUC_0‐inf_ (µg x hr/mL)	C_max_ (µg/mL)	T_max_ (hr)	Reference
Pigs (13 male castrate and 13 female, age ~ 3.4 months, 41.6–54.6 kg BWT, healthy)	8	3 mg/kg, IV	5.89 ± 0.658	—	—	This study
	6	3 mg/kg, IM	3.15 ± 0.696	0.84 ± 0.41^a^	0.083 to 2.50	
	6	6 mg/kg, IM	5.43 ± 0.949	0.96 ± 0.15^a^	0.083 to 2.50	
	6	12 mg/kg, IM	9.60 ± 1.27	1.39 ± 0.44^a^	0.083 to 2.50	
Pigs (12 male; healthy, age not reported, 24.81 ± 1.65 kg BWT)	6	6 mg/kg, IV	3.67 ± 0.75	—	—	Wyns et al. (2014)
	6	6 mg/kg, SC	4.31 ± 1.14	0.41 ± 0.09	0.63 ± 0.21	
Cattle (26, 13 male castrated, 13 female, age < 1 year, 182–260 kg BWT, healthy)	12	3 mg/kg, IV	4.28 ± 0.536	—	—	Huang et al. (2010)
	4	3 mg/kg, SC	4.55 ± 0.690	0.18 ± 0.00^a^	3.3 ± 3.1	
	4	6 mg/kg, SC	9.42 ± 1.11	0.75 ± 0.56^a^	1.0 ± 0	
	4	9 mg/kg, SC	12.2 ± 1.13	0.53 ± 0.12^a^	0.69 ± 0.38	
Cattle (gender, age and BWT not reported; cattle with bovine respiratory disease)^b^	26	6 mg/kg, SC	5.4 ± 0.13	0.13 ± 0.003	—	DeDonder et al. (2016)
Cattle (30; male and female, age 7–8 months, BWT not reported, healthy)	3 × 10	6 mg/kg, SC	7.95	0.433	1.0	Giguère et al. (2011)
Sheep (15; 7 male, 8 female, age 5–6 months, 27.8–38.8 kg BWT, healthy)	15	6 mg/kg, SC	8.00 ± 1.41	0.58 ± 0.17^a^	0.911 ± 1.57	Kellermann et al. (2014)
Foals (3 male, 3 female, age 4–8 weeks, 85–127 kg BWT, healthy)	6	6 mg/kg, IM	4.08 ± 0.455	0.333 ± 0.119	1.0	Berghaus et al. (2012)
Foals (4 male, 6 female, age 6–8 weeks, 105–148 kg BWT, healthy)	10	6 mg/kg, IV	7.00 ± 2.12	—	—	Berlin et al. (2017)
Broiler chicken (12 female, age 4 weeks, BWT 1.369 ± 0.082 kg, healthy)	6 6	6 mg/kg, IV 6 mg/kg, SC	4.00 ± 1.06^a^ 4.10 ± 1.66^a^	−0.89 ± 0.39^a^	−0.13 ± 0.04	Watteyn et al., (2013)
Turkey (64 female, age 3 week, BWT 0.556 ± 0.057 kg)	32 32	6 mg/kg, SC 6 mg/kg, PO	6.85 ± 2.83 2.17 ± 1.30	0.89 ± 0.41 0.087 ± 0.099	0.08 ± 0.00 0.85 ± 0.22	Watteyn et al. (2015)

^a^ transformed from ng/ml to µg/ml.

^b^ only limited blood sampling time points; pharmacokinetic data modelled, IV = intravenous; IM = intramuscular; SC = subcutanous;PO = per os; ‐ = not reported.

Comparable with cattle (Huang et al., 2010), average C_max_ values in swine did not increase proportionally over the dose range 3–12 mg/kg BWT, indicating that the absorption of GAM did not follow linear processes. The rate of absorption from the administration site is limited by blood flow to the injection site and, thus, the maximum plasma concentration did not increase proportionally with a higher dose. Similarly, a slow but continuous release following injection was observed in swine for tylosin and TIL, related 16‐membered semisynthetic macrolide antibiotics (Prats et al., 2002; Rose et al., 2012), which may be attributed to concentration‐dependent plasma protein binding (Rose et al., 2012).

However, as found in cattle with GAM doses of 3, 6 and 9 mg (Huang et al., 2010), approximate dose proportionality was evident in swine for the AUC_inf_ values for doses of 3, 6 and 12 mg/kg BWT. This indicates that the increase in the administered dose was accompanied by a proportional increase in the overall exposure to GAM over the dose range tested. Apart from potential species‐specific physiological differences, absorption in cattle of the 9 mg/kg BWT dose may not be impacted in the same manner as the higher dose of 12 mg/kg BWT in swine. Nonetheless, all doses administered in the present pharmacokinetic study were highly bioavailable (average 93.3%) and absorbed within hours. The rapid absorption within approximately 1 hr following dosing of GAM at 6 mg/kg and high bioavailability of 117.6%, 110% and 102.4%, respectively, has been described in other studies in swine (Wyns et al., 2014), cattle (Huang et al., 2010) and broiler chickens (Watteyn et al., 2013).

### 4.2 Field study

The results of the European multicentre field study show that a single IM injection of GAM 15% w/v at the dose of 6 mg/kg BWT resulted in clinical cure of spontaneously acquired swine respiratory disease associated with *A. pleuropneumoniae* and/or *P. multocida* and/or *B. bronchiseptica and/or H. parasuis* in 97% of the treated swine as demonstrated by the daily observation and scoring of the animals for 10 days after treatment. This has been further supported by the proportion scored acceptable of each of the three clinical parameters which were used to characterize swine respiratory disease (respiratory signs, depression and rectal temperature). A similar level of overall efficacy was seen in the swine treated with the 2011 recently authorized macrolide antibiotic TIL 4% w/v (EMA, 2018b) which was selected as positive control in accordance with the recommendations for the design of non‐inferiority studies (Freise et al., 2013).

No adverse reactions related to treatments were observed during this field study confirming that GAM 15% w/v is a well‐tolerated and safe product for use in swine. Only 6 of the 153 GAM‐treated and 10 of 152 TIL‐treated pigs experienced transient injection site reactions, which resolved rapidly within 1 day after treatment administration and no adverse experiences related to treatment were observed.

This field study was conducted at six commercial farms in three European countries to provide geographical diversity and to allow for the clinical evaluation against swine respiratory disease of GAM under various husbandry conditions with potentially variable infection pressure, and by various investigators. The primary variable for the evaluation of the clinical efficacy against swine respiratory disease of GAM 15% w/v was a combination of scores for respiratory signs and depression, and the rectal temperature to characterize the clinical presentation of swine respiratory disease under field conditions, in line with those recently used to assess macrolide antibiotics in Europe (Nanjiani et al., 2005; Petersen et al., 2012).

Overall, results of this field study are support by recently published studies which reported the use of GAM 15% w/v in the effective treatment of acute outbreaks of *A. pleuropneumoniae* in swine (Papatsiros et al., 2019) and experimentally induced *B. bronchiseptica‐*associated respiratory disease (Gupta et al., 2019).

In conclusion, similar to cattle, pharmacokinetics in swine of GAM are characterized by dose proportionality of AUC, fast absorption, extensive distribution and high bioavailability which are considered beneficial properties for antimicrobial products. The clinical evaluation in the multicentre field study has shown that GAM 15% w/v (Zactran®), administered as a single IM injection, was a safe and efficacious treatment for fattening swine with swine respiratory disease under commercial farming conditions in Europe.

## 5 Ethics statement

All study procedures complied with the appropriate local animal welfare regulations, and were approved by applicable legal bodies and by the company´s animal welfare committees. The on‐farm procedures in the field study were performed with the informed consent of the animal owners.

## AUTHOR CONTRIBUTION

Dietmar Hamel: Conceptualization; Investigation; Writing‐original draft. Alexandra Richard‐Mazet: Conceptualization; Project administration; Writing‐review & editing. Florian Voisin: Investigation; Writing‐review & editing. Inge Böhne: Investigation; Writing‐review & editing. Florence Fraisse: Project administration; Writing‐review & editing. Renate Rauh: Project administration; Writing‐review & editing. Rose Huang: Investigation; Writing‐review & editing. Michael Kellermann: Investigation; Writing‐review & editing. Laura Letendre: Investigation; Writing‐review & editing. Pascal Dumont: Conceptualization; Supervision; Writing‐review & editing. Steffen Rehbein: Conceptualization; Supervision; Writing‐original draft.

## DISCLAIMER

ZACTRAN® is a registered trademark of Merial. All other marks are the property of their respective owners. This document is provided for scientific purposes only. Any reference to a brand or trademark herein is for informational purposes only and is not intended for a commercial purpose or to dilute the rights of the respective owner(s) of the brand(s) or trademark(s). Merial is now a part of Boehringer Ingelheim.

### PEER REVIEW

The peer review history for this article is available at https://publons.com/publon/10.1002/vms3.375.
